# PDJ amplicon in triple negative breast cancer

**DOI:** 10.1038/s41598-023-27887-8

**Published:** 2023-01-12

**Authors:** Alexander S. Roesler, Smriti Malasi, Lori Koslosky, Peter Hartmayer, Tammey J. Naab, Jodi M. Carter, David Zahrieh, David Hillman, Roberto A. Leon-Ferre, Fergus J. Couch, Matthew P. Goetz, Karen S. Anderson, Barbara A. Pockaj, Michael T. Barrett

**Affiliations:** 1 Department of Research, Mayo Clinic in Arizona, Scottsdale, AZ USA; 2 School of Medicine, Duke University, Durham, NC USA; 3 MetaSystems Group, Inc., Newton, MA USA; 4 Department of Pathology, Howard University Hospital, Washington, DC USA; 5 Departments of Laboratory Medicine and Pathology, Mayo Clinic, Rochester, MN USA; 6 Departments of Surgery, Mayo Clinic, Rochester, MN USA; 7 Departments of Health Sciences Research, Mayo Clinic, Rochester, MN USA; 8 Departments of Oncology, Mayo Clinic, Rochester, MN USA; 9 Division of Hematology-Oncology, Mayo Clinic in Arizona, Scottsdale, AZ USA; 10 Biodesign Institute, Arizona State University, Tempe, AZ USA; 11 Division of General Surgery, Section of Surgical Oncology, Mayo Clinic in Arizona, Phoenix, AZ USA; 12 Department of Molecular Pharmacology and Experimental Therapeutics, Mayo Clinic in Arizona, Scottsdale, AZ USA

**Keywords:** Breast cancer, Cancer genomics

## Abstract

Amplification of chromosome 9p24.1 targeting *PD-L1*, *PD-L2*, and *JAK2* (PDJ amplicon) is present in subsets of triple negative breast cancers (TNBCs) and is associated with poor clinical outcomes. However, the prevalence of PDJ+ TNBCs varies extensively across studies applying different methods for interrogating samples of interest. To rigorously assess the prevalence of PDJ amplicons in TNBC, its prognostic value and whether it is enriched by chemotherapy, we interrogated 360 TNBC samples including 74 surgical resections from patients treated in the neoadjuvant setting, and tissue microarrays (TMAs) with 31 cases from African American women and 255 resected non-metastatic cases, with a 3 color fluorescence in situ hybridization (FISH) assay targeting the 9p24.1 PDJ amplicon, 9q24.3, and 9q34.1. Samples with mean PDJ signal of > 4.5 copies, and ratios of PDJ/9q24 ≥ 2 and/or PDJ/9q34.1 ≥ 2 were called amplified (PDJ+). Correlative analyses included the association of tumor infiltrating lymphocytes (TILs) with PDJ amplicons in TNBCs. In addition, we investigated intratumor copy number of PDJ amplicons in PDJ+ and PDJ− TNBCs. Matched pre- and post-neoadjuvant treatment biopsies were available from patients (n = 6) to evaluate the effects of therapy on PDJ status. Our study provides a rigorous analysis of the prevalence, distribution, and clinical correlatives of the PDJ amplicon in TNBC.

## Introduction

There is an emerging recognition of immune checkpoints in the pathogenesis of solid tumors^1^. Immune checkpoints are distinct inhibitory pathways that normally serve to regulate T cell activation and function. These pathways include activation of the programmed cell death protein one (PD-1) receptor by its ligands PD-L1 and PD-L2. Increased expression of PD-1 and its ligands have been reported in human tumors suggesting that tumors may exploit the PD-1 mediated checkpoint to evade immune surveillance^2–5^, augmented by somatic mutational burden^6–8^. Accelerated approval for advanced TNBC was granted to the PD-L1 inhibitor atezolizumab in 03/2019 based on the IMpassion130 trial, and to the PD-1 inhibitor pembrolizumab in 11/2020 based on KEYNOTE-355. Pembrolizumab also has an approved indication for high-risk early-stage TNBC based on KEYNOTE-522. However, the follow-up trial of atezolizumab in advanced TNBC (IMpassion131) failed to demonstrate a survival benefit, such that the manufacturer withdrew its TNBC indication in 08/2021. Nonetheless, evaluation of pembrolizumab in breast cancer is ongoing, with some promising clinical activity in PD-L1 positive estrogen receptor (ER) + breast cancer based on KEYNOTE-028.

Exploratory biomarker analyses suggest an association of clinical response to immune checkpoint inhibitors (ICIs) with the presence of stromal tumor-infiltrating lymphocytes (TILs), CD8 + T cells, and PD-L1 expression, but these associations have yet to demonstrate clinical utility^9^. An emerging picture suggests that tumor specific genomic lesions, either individually or in combination, are associated with immune checkpoint activation and the extent and duration of responses for patients to immunotherapy. These lesions include loss of tumor suppressor genes (*PTEN*), the activation of oncogenic drivers (*EGFR*, *KRAS*, and *PIK3CA*), BRCA mutant and BRCA-like homologous recombination deficient (HRD) genomes, and high mutation burdens including those associated with microsatellite instability (MSI), chromosomal instability (CIN) and aneuploidy^10–16^. Immunohistochemistry (IHC) assays provide the basis for established clinical tests to screen samples for PD-1/PD-L1 activation. However, these assays have variable thresholds with heterogeneous scoring on tumor and immune cells. In contrast, genomic amplifications are specific to tumor cells and provide robust therapy markers (e.g., HER2, FGFR1/2, EGFR), that can be objectively scored in slide-based assays.

The PD-L1 and PD-L2 genes localize to 9p24.1 adjacent to JAK2. We and others have identified a 9p24.1 amplicon with the shortest region of overlap including *JAK2* and *PD-L1* (PDJ amplicon) in a variety of tumors^17–28^. High-level 9p24.1 amplification correlates with activation of the JAK/STAT pathway^29–31^. Overexpression of both JAK2 and PD-L1 mRNA has been associated with 9p24.1 amplification in a *JAK2*-dependent manner^29,30^. The JAK2/STAT3 signaling pathway is required for growth of CD44(+) CD24(−) stem cell-like breast cancer cells, particularly in basal-like breast cancers and is independently associated with breast cancer metastasis and poor prognosis^32,33^. Notably our study of flow sorted tumor populations confirmed that the PDJ amplicon is present in chemoradiation naïve resected cases, includes *JAK2* and *PD-L1*, and correlates with increased RNA expression of JAK2 (p < 0.0001) and of PD-L1 (p < 0.0229)^29^. In addition, our preclinical studies have shown that PD-L1 expression is markedly and rapidly inducible by low-dose IFN-γ in a PDJ amplicon copy-number dependent manner, mimicking an in situ inflammatory response^34^. Thus, rather than constitutive overexpression, the PDJ amplicon is associated with a dynamic cytokine-inducible PD-L1 expression on tumor cells. The prevalence of the PDJ amplicon was found to be ≥ tenfold higher in TNBCs relative to other solid tumors^31^. Notably, the presence of PDJ amplicons has been reported to be limited in newly diagnosed TNBC but enriched by neoadjuvant chemotherapy^30^. These reports highlight a need for additional studies in independent patient cohorts, including pre- and post- neoadjuvant treatment samples to investigate the clinical significance of PDJ amplification in TNBC.

The highly aberrant nature of TNBC genomes and the enriched presence of 9p24.1 amplicons make TNBC a highly favorable tumor model to investigate clinical and genomic correlates of the PDJ amplicon^35^. In order to determine the prevalence of PDJ amplification and its association with clinical variables we developed a robust three color clinical FISH assay that can be applied to routinely collected pathology slides and tissue microarrays (TMAs). In this study we interrogated 360 TNBC cases from three distinct cohorts, an African American cohort (n = 31) from Howard University, surgical resections (n = 74) including 6 cases with matching pre and post neoadjuvant chemotherapy biopsies from Mayo Clinic Arizona (MCA); and treatment naive resected cases (n = 255) from a TMA provided by the Mayo Clinic Breast Cancer Specialized Program in Research Excellence (SPORE). Correlative analyses of the Mayo Clinic TMA results evaluated the association of histopathology and tumor infiltrating lymphocytes (TILs) in those TNBCs with PDJ amplicons. Our study provides a rigorous validation of the prevalence of PDJ amplicons in TNBC with a robust FISH assay across 3 independent cohorts, and a unique analysis of clinical correlates for this subset of TNBCs.

## Results

### Prevalence and heterogeneity of PDJ + cells in TNBC

**Figure 1:**
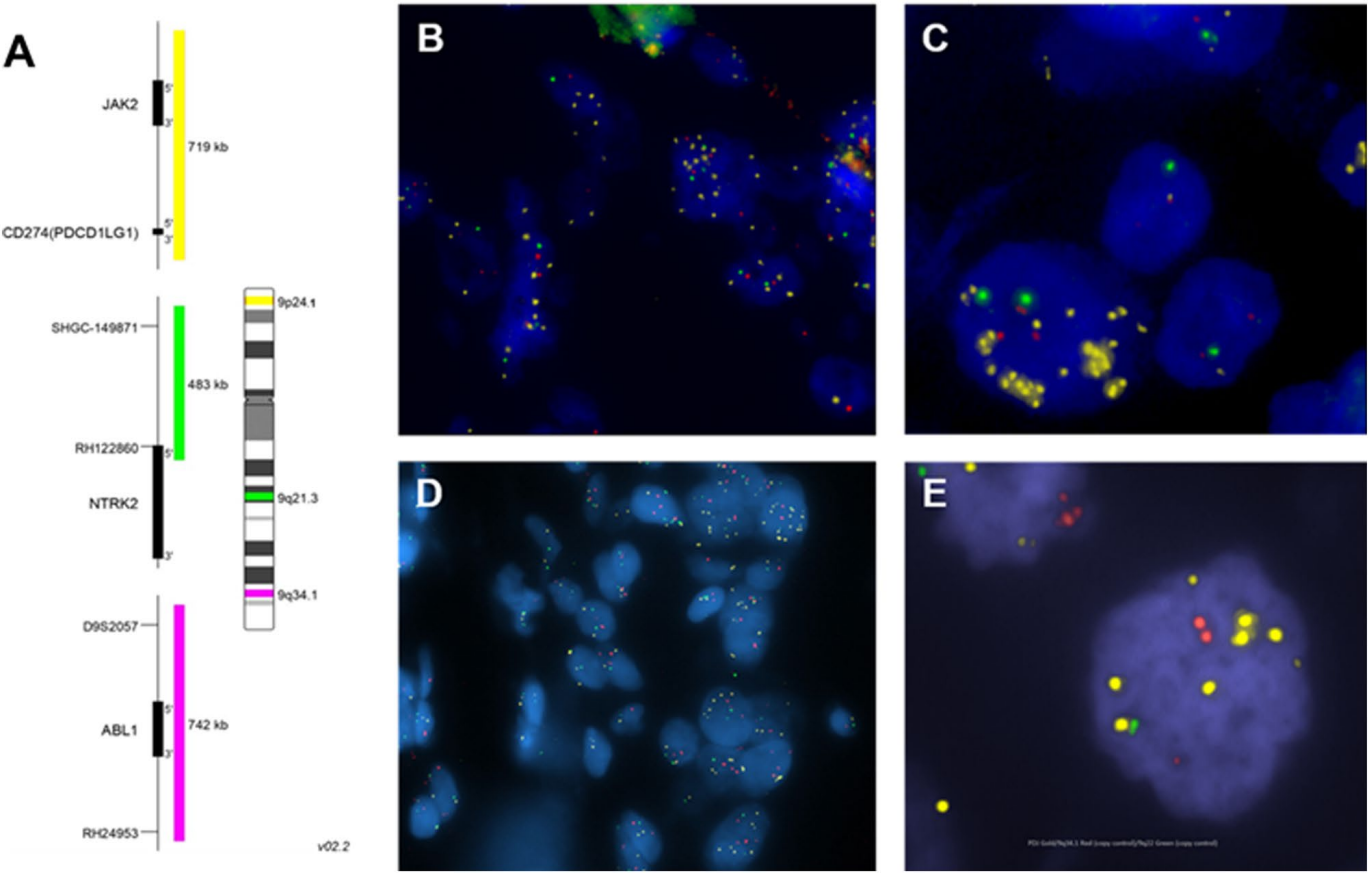
PDJ FISH analysis of TNBC samples. (**A**) Three color FISH assay includes probes for 9p24.1 JAK2/PD-L1 (yellow), 9q21.3 NTRK (green) and 9q34.1 ABL1 (red) that discriminate PDJ amplicons from whole chromosome 9 gains. (**B**,**C**) Mayo Clinic Breast Cancer SPORE TMA1A samples 007 and 094. (**D**) Mayo Clinic Arizona (MCA) resected case MCA PS15.9602. (**E**) Howard University TMA sample DM-2651-A7 from a 30-year-old African American patient**.** PDJ + cases were identified based on our scoring criteria similar to those used for HER2 FISH that include mean copy number of > 4.5 and PDJ/reference ratio ≥ 2.0. In all cases 50 cells were scored to determine the PDJ status.

**Figure 2:**
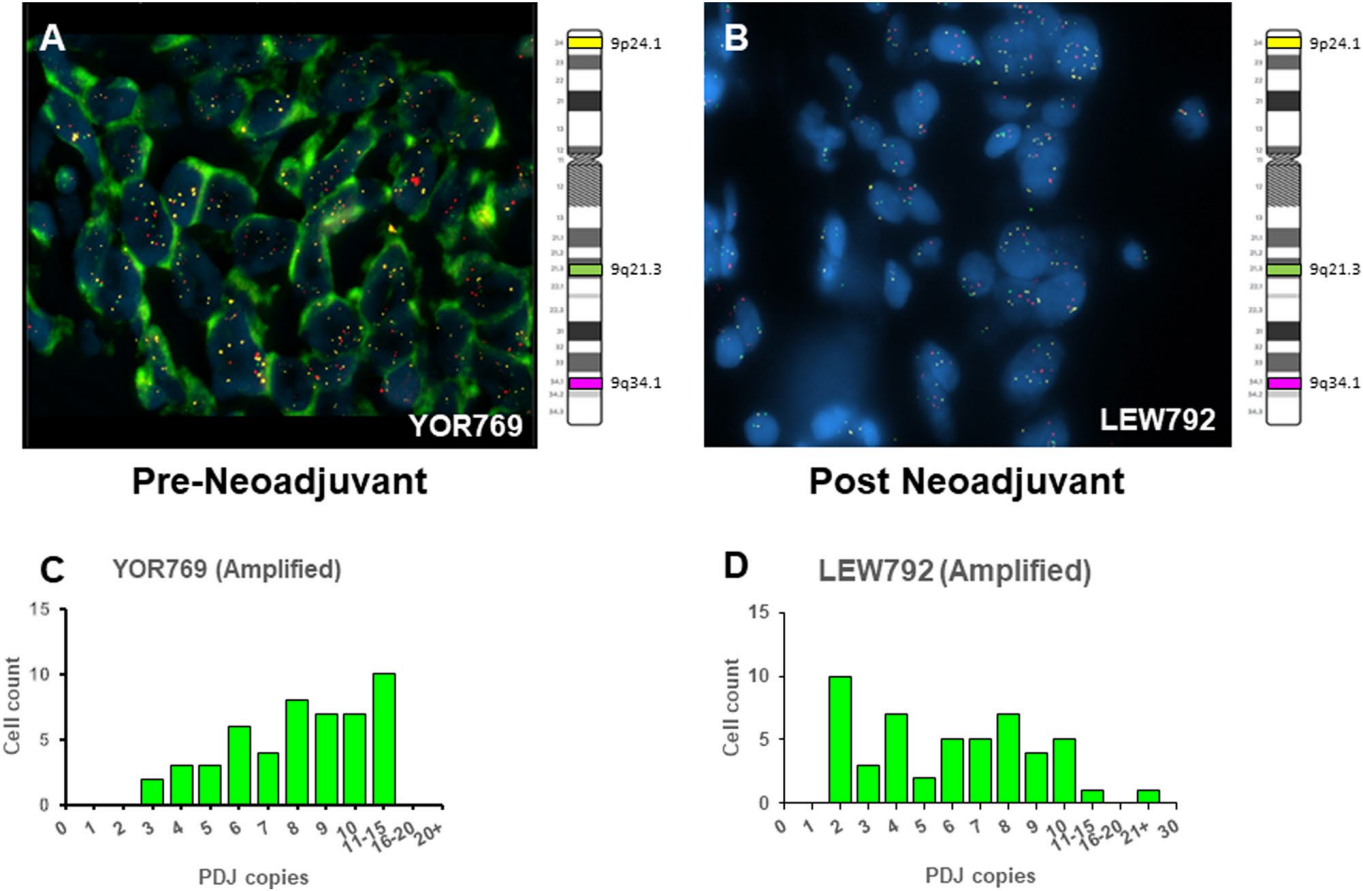
Distribution of PDJ amplicon copies per cell in pre and post neoadjuvant treated TNBC patient biopsies. (**A**,**B**) PDJ copy number was measured by FISH assay across 50 cells per sample in each TNBC biopsy. Average copy numbers ($\overline{{\text{x}}}$) were used to define samples with deletions ($\overline{{\text{x}}}$ < 2), no changes/neutral ($\overline{{\text{x}}}$ = 4), gains (2 < $\overline{{\text{x}}}$ ≤ 4), and amplifications ($\overline{{\text{x}}}$ > 4.5) of PDJ copy number. (**C,D**) Representative examples of JAK2/PDJ signals detected by fluorescence in situ hybridization (FISH) assays.

**Figure 3:**
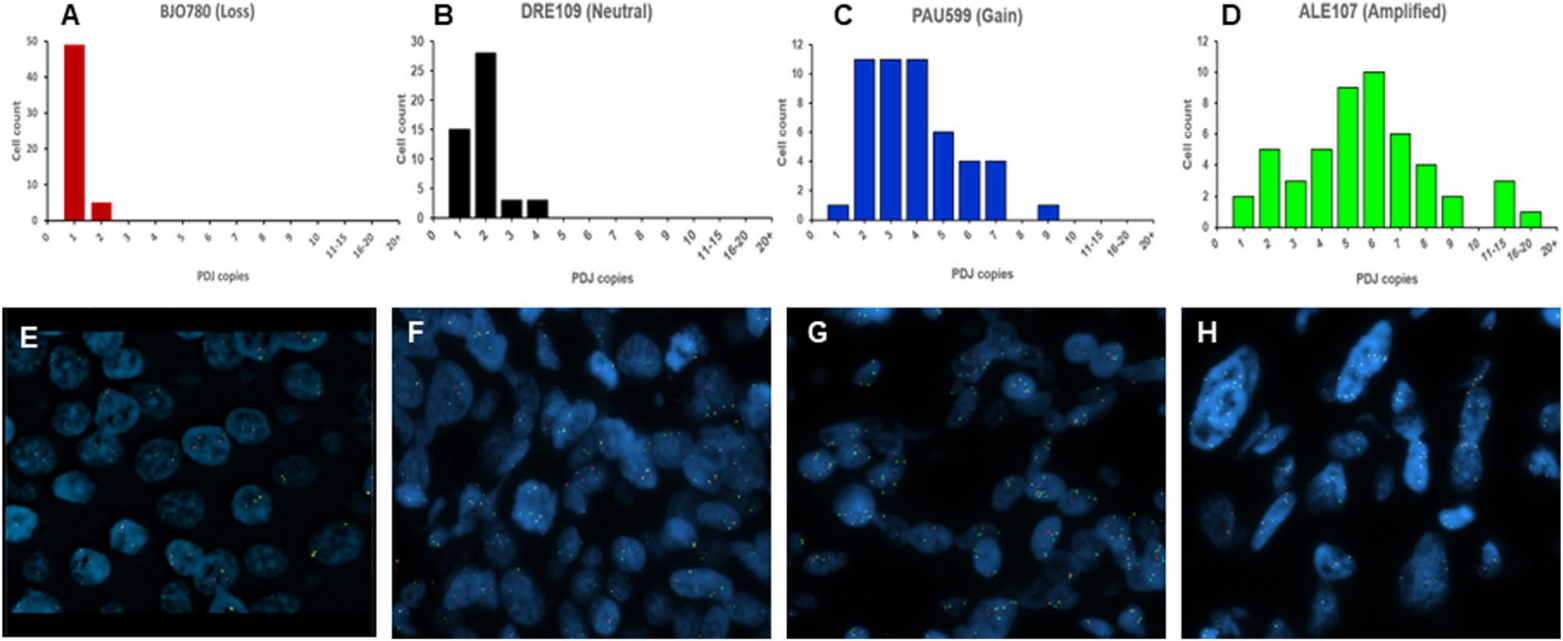
Distribution of PDJ amplicon copies per cell in PDJ- an PDJ + TNBC patient biopsies. (**A**–**D**) PDJ copy number was measured by FISH assay across 50 cells per sample in each TNBC biopsy. Average copy numbers ($\overline{{\text{x}}}$) were used to define samples with deletions ($\overline{{\text{x}}}$ < 2), no changes/neutral ($\overline{{\text{x}}}$ = 4), gains (2 < $\overline{{\text{x}}}$ ≤ 4), and amplifications ($\overline{{\text{x}}}$ > 4.5) of PDJ copy number. (**E**–**H)** Representative examples of JAK2/PDJ signals detected by fluorescence in situ hybridization (FISH) assays.

We screened a total of 360 TNBC cases from the three distinct cohorts of patients with our three color FISH assay (Fig. 1). The first cohort included 97 samples from 74 resected TNBC patients, all of whom received neoadjuvant chemotherapy prior to surgery. Clinical features of the neoadjuvant TNBC cohort include smaller tumor sizes (75.7% T1–T2 vs 24.3% T3–T4), reduced lymph node involvement (24.3% positive vs 75.7% negative), early stage of disease (74.3% Stages 1–2 vs 25.7% Stages 3–4) and high-grade pathologies (78.4% G3 vs 20.2% G1–G2) (Table 1). Thirty-four of the samples were obtained prior to neoadjuvant therapy, including biopsies of six cases with patient matched resected samples. The remaining samples were obtained from the post treatment surgical resections. We benchmarked our FISH assay with array comparative genomic hybridization (aCGH) results of flow sorted TNBC tissue (n = 14) from the MCA cohort^29^. For example, MCA TNBC-11 had a high level (*x̅* > 15 copies/cell, aCGH log_2_ratio > 4) 9p24.1 copy number with low level gain (2.5 copies/cell) in the pericentromeric 9q21.3 region, and diploid copies at 9q34.1 (Supplemental Fig. 1A,B). In contrast MCA TNBC-9 had loss (*x̅* < 1.8 copies/cell,  aCGH log_2_ratio < 0.7) at 9p24.1 and 9q34.1, with low level gain of 9q21.3 (Supplemental Fig. 1C). Nine of 74 patients (12.2%) in our neoadjuvant cohort were positive for PDJ amplification including treatment naïve samples from four patients (Fig. 2A, Table 2). In addition to the PDJ + cases, we identified 22 tumors (29.7%) with PDJ copy number gain including nine with gain of whole chromosome 9, 36 tumors (48.6%) with neutral copy number, and 7 tumors (9.5%) with deletions of 9p24.1 (Fig. 3).

**Table 1:** Neoadjuvant TNBC Cohort.

Clinical characteristics	Total
	Patients (n = 74) (%)
Age (years)	
Average	57.3
Median	59
Range	26–82
Tumor size	
T1–T2	55 (74.3)
T3–T4	18 (24.3)
Unknown	1 (1.4)
Lymph node status	
Positive	17 (22.9)
Negative	55 (74.3)
Unknown	2 (2.7)
Tumor stage	
1–2	54 (72.9)
3	18 (24.3)
4	1 (1.4)
Unknown	1 (1.4)
Pathology grade	
G1–G2	15 (20.2)
G3	58 (78.4)
Unknown	1 (1.4)
Neoadjuvant therapy	
Yes	74 (100)
No	0 (0)
Unknown	0 (0)
PDJ copy number	
Amplified	9 (12.2)
Gain	22 (29.7)
Neutral	36 (48.6)
Deletion	7 (9.5)

**Table 2:** The PDJ FISH.

TNBC Cohort	Total	PDJ +
Neoadjuvant MCA	74	9 (12.1%)
Howard TMA	31	6 (19.3%)
Mayo Clinic Breast Cancer SPORE TMA	255	36 (14.1%)
Combined	360	51 (14.1%)

The distribution of PDJ amplicon copies per cell in the TNBC biopsies was found to vary significantly with increasing mean copy number status (Fig. 3). Tissues with PDJ deletions (x̅ < 2) or neutral copy numbers (x̅ = 2) tended to exhibit narrow ranges (0–3 copies) of PDJ amplicon copies per cell. In contrast, tumors with PDJ copy number gains or amplifications were found to have broader distributions that included individual cells with 10 or more copies in the same sample. The nine positive cases included cells with as many as 26 copies and up to 17/50 (34%) cells with ≥ 10 copies in each PDJ + sample (Fig. 2C,D). Images from the FISH analyses were examined to assess the spatial distribution of PDJ amplicons in TNBC tissues with increased copy numbers (Figs. 1, 2, 3). The PDJ + TNBCs displayed remarkable heterogeneity with cells possessing ≥ 15–20 PDJ copies located immediately adjacent to tumor cells with only 1–2 copies. In addition to the PDJ + biopsies, three PDJ gain samples (2 post and 1 pre neoadjuvant biopsies) had at least one cell with ≥ 10 copies of PDJ amplicon.

In the African American (AA) cohort, ages ranged from 28 to 76 (average 47), and stage at diagnosis ranged from I-IV. We obtained results from a single TMA slide that passed quality control including a series of diploid normal samples. Scoring was targeted to 20 intact cells per case. Six of 31 (19%) tumors on the slide had PDJ amplification by FISH, of which five were from breast tissue and one was from a lymph node (Fig. 1E, Table 1). PDJ + cases included cells with up to 14 copies, with an additional PDJ gain case containing a cell with 11 copies.

The third cohort was a TMA containing surgically treated stage I-III TNBCs (Table 3)^36^. We obtained FISH data from 255 cases on the TMA including 143 cases with replicate spots that could be read and 112 with results from a single spot on the array. Thirty-six of 255 (14.1%) evaluable cases were PDJ + based on our scoring criteria (Table 2). Similar to our cohort of resected cases the distribution of PDJ + cells within each positive case included cells with as many as 30 copies and up to 28/50 (56%) cells with ≥ 10 copies in each PDJ + sample (Fig. 1B,C). Correlative analyses of results from the Mayo Clinic Breast Cancer SPORE TMA identified an association of increased TILs in those TNBCs with PDJ amplicons (Wilcoxon Rank Sum 2-sided p-value = 0.01) (Table 4). The median stromal TIL counts for the PDJ positive and PDJ negative groups were 30 and 20.0, respectively.

**Table 3:** The Mayo Clinic Breast Cancer SPORE TMA.

	PDJ		P-value
	Positive (N = 36)	Negative (N = 222)	
Menopausal status, n (%)			0.7251^1^
Pre	13 (36.1%)	87 (39.2%)	
Post	23 (63.9%)	135 (60.8%)	
Age, n (%)			0.1243^1^
LT 50	9 (25.0%)	85 (38.3%)	
GE 50	27 (75.0%)	137 (61.7%)	
KI-67 group, n (%)			0.0572^1^
At most 15%	2 (5.6%)	46 (20.8%)	
15.1–30%	9 (25.0%)	34 (15.4%)	
More than 30%	25 (69.4%)	141 (63.8%)	
Missing	0	1	
Tumor size, n (%)			0.2725^1^
0.1–2.0 cm	13 (36.1%)	111 (50.0%)	
2.1–5.0 cm	21 (58.3%)	98 (44.1%)	
5.1 + cm	2 (5.6%)	13 (5.9%)	
Nodal group, n (%)			0.3657^1^
N0	18 (50.0%)	145 (65.3%)	
N1	12 (33.3%)	49 (22.1%)	
N2	3 (8.3%)	15 (6.8%)	
N3	3 (8.3%)	10 (4.5%)	
NX	0 (0.0%)	3 (1.4%)	
Nottingham grade, n (%)			0.3204^1^
Grade 1–2	1 (2.8%)	16 (7.2%)	
Grade 3	35 (97.2%)	206 (92.8%)	
Stromal tils, n (%)			0.0325^1^
0–10%	4 (11.1%)	77 (35.2%)	
10–20%	9 (25.0%)	48 (21.9%)	
20–40%	11 (30.6%)	47 (21.5%)	
> 40%	12 (33.3%)	47 (21.5%)	
Missing	0	3	
Histology, n (%)			0.3807^1^
Invasive	25 (69.4%)	139 (62.6%)	
Medullary	9 (25.0%)	46 (20.7%)	
Metaplast	1 (2.8%)	23 (10.4%)	
Aprocrine	1 (2.8%)	14 (6.3%)	
Mastectomy, n (%)			0.0504^1^
Mastectomy	20 (55.6%)	85 (38.3%)	
Lumptectomy	16 (44.4%)	137 (61.7%)	
Adjuvant RT, n (%)			0.5236^1^
Yes	18 (60.0%)	126 (66.0%)	
No	12 (40.0%)	65 (34.0%)	
Missing	6	31	
Adjuvant chemo, n (%)			0.0410^1^
Yes	28 (87.5%)	136 (70.1%)	
No	4 (12.5%)	58 (29.9%)	
Missing	4	28	

^1^Chi-Square p-value.

**Table 4:** Analysis variable: stromal TILS.

PDJ	N	Mean	Median	Lower Quartile	Upper Quartile	Range
0	219	26.89	20.00	10.00	40.00	1.00–90.00
1	36	35.86	30.00	15.00	55.00	1.00–80.00

PDJ positivity was associated with higher stromal tils counts (Wilcoxon Rank Sum 2-sided p-value = 0.01).

### PDJ amplicon and chemotherapy

There were seven patients from our neoadjuvant cohort with matching pre- and post-treatment biopsies. FISH results were obtained from six of the matching pairs that included variable baseline PDJ copy numbers (Table 5). In none of these cases did we observe an enrichment of the PDJ amplicon in the post treatment sample. Rather the PDJ copy number was stable in 5/6 of these cases and decreased in one amplified case.

**Table 5:** Pre and post neoadjuvant TNBC.

TNBC	PDJ Copies (Mean)		Neoadjuvant therapy
	Pre	Post	
REI778	1.98	2.1	Paclitaxel followed by doxorubicin + cyclophosphamide
MAR694	1.94	1.96	Carboplatin + paclitaxel
YOR769	8.24	3.28	Docetaxel + carboplatin followed by doxorubicin + cyclophosphamide
COL783	1.04	Fail	Paclitaxel + carboplatin
SHA107	5.58	5.88	Paclitaxel + carboplatin followed by doxorubicin and cyclophosphamide
SBR499	2.54	1.92	Doxorubicin + cyclophosphamide followed by paclitaxel + carboplatin
MCV761	1.85	1.8	Paclitaxel + carboplatin followed by doxorubicin + cyclophosphamide

## Discussion

TNBC is an aggressive disease with poor clinical outcomes. Thus, there is a need to identify markers that can be exploited for improved clinical care. We and subsequently others have identified the PDJ amplicon as a clinically relevant driver of aggressive TNBC. Prior studies assessing resections of primary TNBC tissues have associated PDJ amplification with increased tumor size, frequent lymph node involvement and advanced disease (Stages 3–4), and reduced progression free survival (PFS) and overall survival (OS)^29,30^. Furthermore, there is emerging data suggesting that the presence of the amplicon is associated with increased JAK2/pSTA3 signaling and a reprogrammed immune environment. Immune checkpoint blockade with anti-PD-L1 therapies that overcome tumor-mediated local immunosuppression have been shown to induce regression in 13–38% of metastatic TNBCs that are PD-L1 + by immunohistochemistry (IHC) staining^29–31^. Response to ICIs in TNBCs has been associated with tumor PD-L1 expression^37^. However, the significant temporal and spatial heterogeneity of PD-L1 expression seen with IHC complicates its use as a robust biomarker to identify TNBC patients likely to respond to ICI therapies^38–40^.

Genomics-based biomarkers of PD-L1 expression may provide alternative or complementary means of addressing tumor heterogeneity and identifying cohorts of TNBC patients likely to respond to ICI therapies. The PDJ amplicon is associated with tumor PD-L1 expression in a dynamic, copy-number dependent manner and is regulated by active JAK-STAT signaling^32,34,41^. Our preclinical studies have shown that PD-L1 expression is markedly and rapidly inducible by low-dose IFN-γ in a PDJ amplicon copy-number dependent manner, mimicking an in situ inflammatory response. Thus, rather than constitutive overexpression, the PDJ amplicon is associated with a dynamic cytokine-inducible PD-L1 expression on tumor cells that may correlate with response to PD-L1 inhibition. Notably, our previous studies have shown that PD-L1 protein expression is highly variable within PDJ + tumors with a striking difference in staining intensity between the undifferentiated regions of the tumor (IHC score 5) and those that were differentiated (IHC score 0)^42^. Of significant interest in future studies will be to assess whether PDJ amplifications as measured with our FISH assay, can predict response to ICI therapies regardless of the level of PD-L1 expression measured by IHC^41,43,44^.

Our neoadjuvant TNBC cohort consists of 97 biopsies from 74 patients from a single institution (MCA). The median age of patients in this cohort was 59 with an enrichment for smaller (74.3% T1-T2) and earlier stage (72.9% 1–2) tumors. Notably, this cohort included six cases with matching pre- and post-treatment biopsies. The PDJ amplicon was noted to have broader distributions with 1–20 PDJ copies per single tumor cell in PDJ-amplified patients, which is consistent with the high degree of heterogeneity seen with PD-L1 expression in TNBCs. Strikingly, three PDJ gain patient samples were observed to have at least one cell with ≥ 10 PDJ copies. The clinical significance of these PDJ + cells in otherwise PDJ- tumors remains to be determined.

TNBC disproportionately affects young African American women^45^. In addition, AA women with TNBC have worse clinical outcomes than women of European descent. However, it remains to be determined whether this is due to distinct molecular features of disease or to socioeconomic factors including disparities in access to health-care treatment, co-morbid disease, and income. Although limited in the number of evaluable cases, the prevalence of PDJ + cases in the AA cohort was similar to that from the surgical resection cohort and the Mayo Clinic Breast Cancer SPORE TMA of untreated TNBCs. Larger follow up studies with additional cases from AA women will provide a more complete profile of PDJ amplicons in this population.

The presence of TILs has been reported in patients who responded to ICIs in several clinical trials. For example, in IMpassion130, joint analysis of PD-L1 and TILs found that cases which were PD-L1 positive (SP142) and had TILs > 10% had outcomes which appeared superior to cases which were PD-L1 positive alone as per the primary analysis^46^. Our results from the Mayo Clinic Breast Cancer SPORE TMA identified an association of increased stromal TILs in those TNBCs (14%) with PDJ amplicons targeting PD-L1 and JAK2 (P < 0.01).

The limitations of this study listed include the lack of detailed genomic characterization of the patient samples and post-surgical clinical outcomes (Table 6). The analysis of matching pre and post neoadjuvant treated samples was limited to 6 pairs. In addition, our cohorts do not contain biopsies from patients treated with ICIs. However, our survey of three distinct cohorts of TNBC cases establishes a baseline prevalence (14%) for PDJ + in this aggressive subtype, includes scoring of single cells within each tumor, and provides preliminary correlative association with increased TILs, a signature associated with improved responses to ICI therapies.

**Table 6:** Strengths and weaknesses.

Strengths	Weaknesses
Validated 3 color FISH assay	Focussed on chromosome 9
Single cell scoring	No additional genomic data (e.g., mutations, copy numbers) for TMA samples
Three independent cohorts	No ICI treated samples and response data
Extensive clinical annotation with Mayo Clinic Breast Cancer SPORE TMA	No ICI treated samples and response data
Matching per and post neoadjuvant biopsies	Limited to 6 cases

We validated our FISH assay with aCGH results from a subset of flow sorted TNBCs in the surgical cohort (Supplemental Fig. 1). Furthermore, our 3 probe design enabled the discrimination of PDJ amplifications from whole chromosome 9 aneuploidies that are prevalent in TNBC. The height of this amplicon in our studies includes aCGH log_2_ratios > 4 corresponding to FISH copy numbers > 20 consistent with amplification of genomic “drivers” such as *HER2* and *MYC* in breast cancer and other solid tumors. Notably, single cells with ≥ 10 copies were observed in each PDJ + sample and in a subset of PDJ- TNBCs. Thus, our preliminary observations support evidence from prior studies that suggest low frequency PDJ + cells are present in a subset of non PDJ amplified TNBCs^30^. However, contrary to previous studies we did not observe an enrichment of PDJ + cells in the neoadjuvant chemotherapy setting^30^. Larger studies, including those with multi regional biopsies from individual patients, will be required to resolve whether PDJ + cells are enriched in the neoadjuvant setting. Furthermore, what role, if any, individual tumor cells with high PDJ copies might have in otherwise PDJ-negative TNBCs has yet to be determined.

### PDJ amplification and immune cells in TNBC

Our results from the Mayo Clinic Breast Cancer SPORE TMA identified an association of increased stromal TILs in those TNBCs (14%) with PDJ amplicons targeting PD-L1 and JAK2 (P < 0.01). The median stromal TIL counts for the PDJ positive and PDJ negative groups were 30 and 20.0, respectively. Notably the presence of TILs has been reported in patients who responded to ICIs in several clinical trials. For example, in IMpassion130, joint analysis of PD-L1 and TILs found that cases which were PD-L1 positive (SP142) and had TILs > 10% had outcomes which appeared superior to cases which were PD-L1 positive alone as per the primary analysis. TILs alone also predicted benefit from the addition of atezolizumab. Our hypothesis is the PDJ amplicon is a robust genomic marker for those TNBC patients likely to respond to ICI. Given direct associations of PDJ + with PD-L1 and stromal TILs, our initial focus will be patients treated with a PD-L1 inhibitor (atezolizumab). However, we are aware of the use and clinical benefit of PD-1 inhibitors (pembrolizumab) in early stage and metastatic TNBC so evaluation will extend to these patient populations in future studies.

### Evidence for a clinical impact of 9p24.1 amplification in other tumor types

In lymphoma, JAK2 has been shown to up regulate the transcription of both PD-1 ligands (PD-L1, PD-L2) while increasing sensitivity to JAK2 inhibitors in a dose dependent manner^25^. In addition, there have been case reports of significant responses to immune checkpoint inhibition in solid tumors with 9p24.1 amplicons targeting JAK2, PD-L1 and PD-L2^47–49^. Notably these occurred in advanced microsatellite stable tumors including non-small cell lung cancer, a colon adenocarcinoma, and a cancer of unknown primary origin. There is also emerging data that a 9p24.1 amplicon is present in Epstein-Barr virus (EBV)-positive gastric cancers and in squamous cell carcinoma (SCC) of the cervix or vulva^25,27,50^. In our preliminary published data, we found the PDJ amplicon in a subset (3–5%) of glioblastomas (2/44) and colorectal cancers (2/68)^29^. Similar findings have been reported in these and other solid tissue tumors^26,43,51^. Although relatively rare the presence of PDJ amplicons in common tumor types such as lung and colorectal cancers highlight the potential clinical impact of this genomic lesion in addition to TNBC.

## Materials and methods

### Clinical samples

All patients gave informed consent for collection and use of the samples. All tumor samples were histopathologically evaluated prior to genomic analysis. All research was performed in accordance with relevant guidelines/regulations and conformed to the Helsinki Declaration (https://www.wma.net/policies-post/wma-declaration-of-helsinki-ethical-principles-for-medical-research-involving-human-subjects/).

### MCA surgical resection cases

TNBC samples were obtained under a Mayo Clinic protocol 2130-00 Cancer Tissue Study (Principal Investigator Dr. B. Pockaj). This study was approved by Mayo Clinic IRB protocol 08-006579-08 Breast Cancer Clinical Genomics Project. Estrogen receptor (ER) and progesterone receptor (PR) were evaluated by standard ASCO/CAP guidelines with < 1% of the cells staining for the receptors respectively^52^. HER2 negative was defined by ASCO/CAP guidelines as staining by IHC of 0 or 1 + ^53^. HER2 IHC of 2 + was further evaluated by FISH and deemed negative by standard ASCO/CAP guidelines.

### Howard University TMA

A unique TMA representing 124 AA TNBC patients with invasive ductal carcinoma with duplicate cores for each was available. There is normal breast tissue on the TMAs with replicate spots. The cases range from diagnosis in 1998 to diagnosis in early 2013 with > 5 year follow up on 121/124 cases. The cases range from diagnosis in 1998 to diagnosis in early 2013 with > 5 year follow up. A subset of 31 cases in this cohort from a single slide were evaluable in this study.

### MCR TMA

The construction and content of the Mayo Clinic Breast Cancer SPORE TMA is described in previous publications^36,54^. Briefly, it contains tissues from a cohort of 605 women who met the criteria for TNBC (ER/PR\1% and HER2 negative) with surgically treated non-metastatic breast cancer. All patients with clinically reported ER-negative/borderline (B10%) disease were selected for central assessment of ER/PR/HER2, histopathology, Ki-67, and TILs as previously described in studies with this TMA^36,54,55^. The TMA consists of stage I-III breast cancer (BC) samples from patients who underwent surgery between January 1, 1985, and December 31, 2012, and who were clinically HER2 negative or unknown and did not receive anti-HER2 therapy. Patients with prior cancer, bilateral BC, metastatic disease within 60 days of surgery, non-invasive or benign breast disease only, receipt of adjuvant endocrine therapy, known ER > 10% or ER-negative or unknown who received any neoadjuvant therapy were excluded from this cohort.

### Fluorescence in situ hybridization (FISH)

#### Three color assay

The clinical samples and the TMAs were interrogated with a multicolor FISH assay consisting of three chromosome 9 probes that simultaneously target *JAK2* and *PD-L1* (Tamra), chromosome 9 peri-centromere (SpectrumGreen), and 9q34.1 (TexasRed) regions (Supplementary Fig. 1). Each probe consisted of two non-overlapping bacterial artificial chromosome (BAC) clones that mapped to the three loci of interest. This enables discrimination of PDJ amplicons from chromosome 9 ploidies that can occur in the background of genomic instability in aneuploid cancer genomes. The enumeration probe set was applied to individual slides, hybridized, and washed according to published protocols^56^. FISH array imaging and scoring for the 74 TNBC clinical cases and the MCR TMA were performed by Mayo Clinic Cytogenetics Core with scoring of 50 cells for each sample performed by two experienced cytogenetic technicians. The AA sample TMA was imaged and scored by MetaSystems Group, Inc. (Newton, MA, USA) using their MetaFer slide scanning and MetaCyte FISH platform. For each cohort PDJ status was determined based on HER2 scoring guidelines according to the American Society of Clinical Oncology (ASCO)/College of American Pathologists (CAP)^57^. Specifically, PDJ amplification was called in samples with an average copy-number > 4.5, a ratio of PDJ to 9q24.3 (peri-centromere) and/or PDJ to 9q34.1 ≥ 2.0, and at least one cell with ≥ 10 + PDJ copies. PDJ gain was defined as an average copy number 2 < x ≤ 4.0, and a ratio of PDJ to 9q24.3 or to 9q34.1 ≥ 2.0. Copy number neutral status was identified as average PDJ copies of 2.0, while PDJ deletions were all average copy numbers < 2.0.

### Statistical analysis

Statistical correlations of oncogene mutation frequencies and clinical characteristics between cohorts were performed with Pearson’s chi-square test for categorical variables and unpaired Student’s *t*-tests for continuous variables. Comparisons of aCGH and fluorescence in situ hybridization assays used Pearson’s chi-square tests. Pearson correlation coefficients ≤ 0.05 were considered significant. Unpaired Student’s *t*-tests were conducted for average copy number per cell and for average PDJ/chromosomal probe copy number ratios. Statistical significance was defined as *p*-values ≤ 0.05. All statistical analyses were performed using GraphPad Prism 8.

## Supplementary Information

Supplementary Figure 1.

## Data Availability

All aCGH data discussed in this publication have been deposited in NCBI's Gene Expression Omnibus^58^ and are accessible through GEO Series accession number GSE107764.
