# Supplementary figures and images for: PDJ amplicon in triple negative breast cancer
Source: Sci Rep. 2023 Jan 12;13:618. doi: 10.1038/s41598-023-27887-8 (PMC9837184; doi:10.1038/s41598-023-27887-8)

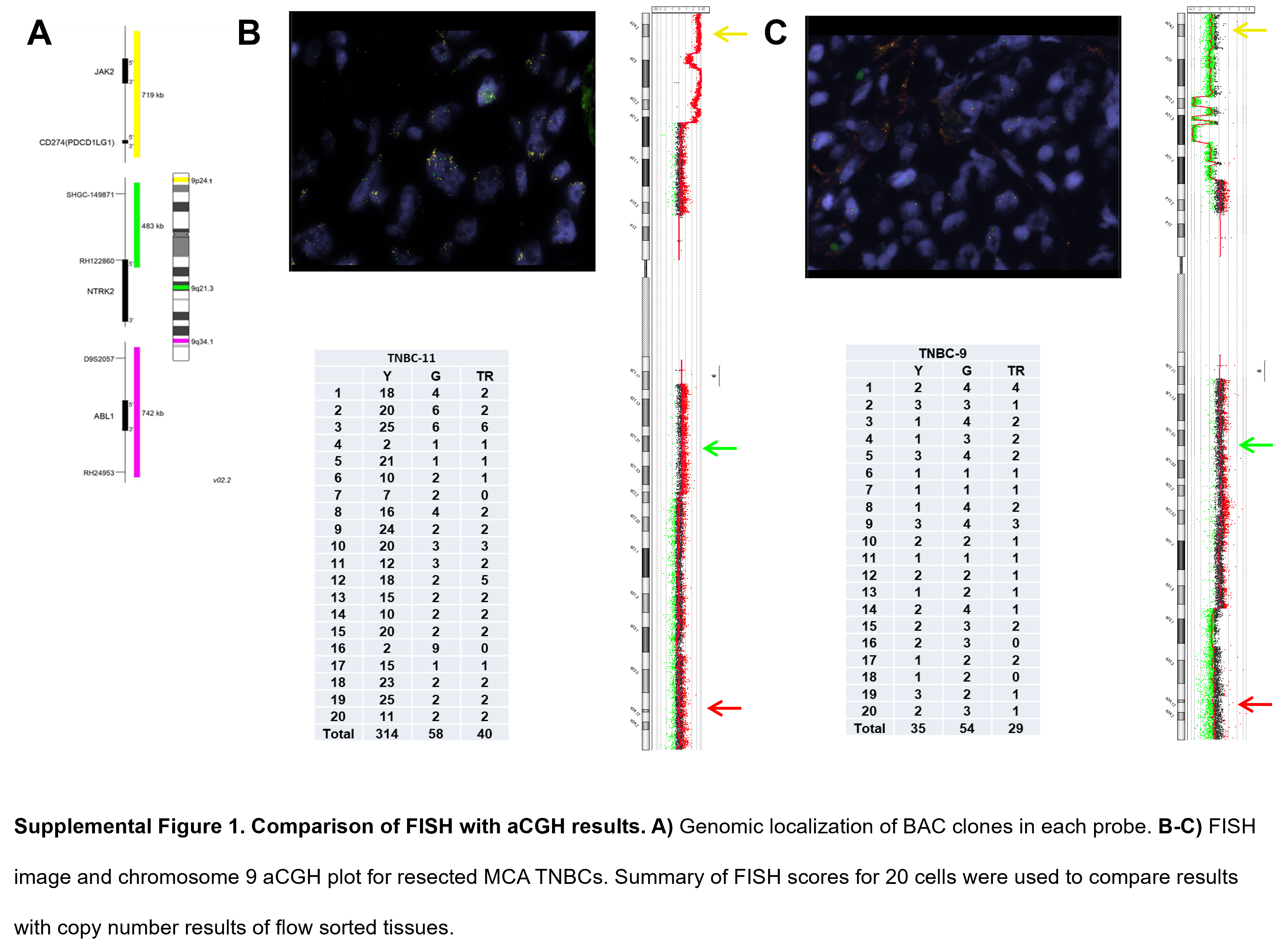

Supplement: Supplementary file 1 — Supplementary Figure 1. [file 41598_2023_27887_MOESM1_ESM.tif]
